# The resilience of public science and effectiveness of policy handling of technical advice

**DOI:** 10.1016/j.fhj.2024.100024

**Published:** 2024-03-05

**Authors:** Christopher JM Whitty

**Affiliations:** Department of Health and Social Care, 39 Victoria Street, London SW1, UK

The extraordinary improvements in medicine which have occurred over the last 150 years have been rooted in clinical innovation and scientific discovery. Central to any effective medical response in both normal and emergency settings is having the best available scientific evidence and advice, whether to patients, healthcare leaders or political leaders.

Even outside emergencies there are however common beliefs amongst people who have leadership positions including in health services that stand in the way of effective deployment of science and undermine its resilience. These can be broadly divided into barriers to undertaking research; barriers to uptake of scientific information by healthcare professionals; barriers to uptake of scientific advice by political and other leaders who are usually not trained in clinical sciences. It is important to respect the intentions of those who hold these beliefs, even when they are standing in the way of an effective science-led clinical response, as those holding them are invariably aiming to do their best for their patients and the general public. We still need however to address them, whether held within the medical profession or outside it.

## Science in emergencies: barriers to research

The COVID-19 pandemic is the latest major emergency which demonstrated the power of science rapidly to protect health in the face of new threats, in addition to the proven ability of research incrementally to reduce morbidity and mortality from established diseases. Initially advice, whether to patients, politicians or the profession had to be based on scientific first principles as this was a new infection. The speed at which research was conducted was remarkable, and the UK played a very major part in this; soon advice could be based on information specific to this new disease, and then effective medical countermeasures including drugs, diagnostics and vaccines were rapidly produced and tested at scale. The scientific response to HIV, the last pandemic of a similar scale, was equally impressive, substantially de-risking a disease with initially 100% mortality, but took longer.^1^ In both cases the new science led to rapidly falling mortality.

The evidence that effective emergency medical responses have always needed to be rooted in strong science, whether old science repurposed to current situation or new science developed in response to changing circumstances is objectively overwhelming. This should sound obvious, but it is not intuitive to sensible and well-meaning people who have been trained in different traditions. Several perfectly reasonable arguments tend to be believed and deployed which need to be taken seriously if we are to defend the integrity and centrality of science. The two most common are:

Argument 1: ‘Not now- can't you see there is an emergency on’. People leading or engaged in emergency responses frequently respond to the suggestion that what is needed now is some research with scepticism, occasionally bordering on incredulity. They usually are prepared to take into account existing science, but with a multiple competing demands on time and resource the idea that scientists should be brought in to do research seems to them incongruous. The perfectly understandable, but incorrect, assumption is that this will only take key people and resources away from the necessary response. It is in part for this reason that many key questions in emergency and humanitarian response from pandemics to tsunamis have never adequately been tested or answered. The reality is that just as with all other areas of medicine if we do not test out our understanding and the effectiveness of existing solutions there is a high chance that we will continue to do things, in good faith, that are less effective than we thought, entirely pointless or in some cases harmful. We will then repeat our previous mistakes.

Argument 2: ‘People are dying, something must be done urgently, this is something, get on and do it’. COVID-19 demonstrated the strong and entirely understandable desire in the face of an emergency appeal just to get on and do stuff. Several drugs were very strongly promoted early in the COVID-19 pandemic, with varying degrees of logic behind them but without any clinical evidence of efficacy. An example of this was chloroquine. It was a perfectly reasonable thing to consider whether it might have some use, and to test that in clinical studies, but the urgency of the situation prompted many non-clinicians (and a few clinicians as well) to say that clinical trials were unnecessary given the scale of the emergency and its speed of onset. This was resisted in the UK where clinicians were extremely disciplined about randomising people into clinical trial such as RECOVERY, which demonstrated that chloroquine did not have any benefits for most people with COVID-19. Worldwide however this urge to use the drug in advance of clinical evidence because it was an emergency may well have cost lives.^2^ All the drugs and other treatments proposed for COVID-19 have potential side-effects which are only justified if the benefits outweigh them. It is essential that clinicians stick to the principle of undertaking trials of new treatments even in the face of very clear and urgent need.

## Science outside emergencies: barriers to research

It is not however only in infectious emergencies that clinical science has recently transformed the outlook for populations and individual patients; the rapid reduction in child mortality globally,^3^ the substantial decrease over the last four decades in cardiovascular mortality^4^ and morbidity and current improvements in cancer care are also obvious examples.

Again many of the arguments are predictable. They are held in good faith by people who want the best for patients, citizens and the health service and need to be taken on bearing that in mind. In terms of barriers to essential research being undertaken three arguments are worth highlighting, of which two tend to be deployed by people working in the health service.

Argument 3. ‘My hospital/department/speciality/general practice is too busy to be undertaking research; I can't afford to have any clinician spending time on peripheral activities when the system is under such stress’. This argument has both short-term and long-term flaws. The long-term one is the most obvious; any discipline which fails to undertake research is destined to improve more slowly for future patients than disciplines which do, or not improve at all. Much of this is actually custom and practice rather than following clinical logic. Some disciplines have a tradition of a much higher proportion of staff being research active to some degree such as oncology, neurology, infectious disease or cardiology. Others with equally able clinicians do not, including care of the elderly and paediatrics. The short-term arguments against however are just as important. It is essential that all doctors and other healthcare workers have some variety in their professional lives, to retain interest and reduce the risks of burnout. For some this will be research, for others teaching, others again may be involved in leadership or administration. If a clinician who wishes to undertake research is stopped from doing so as a minimum they will become demotivated and many will move to other places which do allow them to follow their interests. It is therefore extremely short-term thinking to assume that preventing them from undertaking research will actually improve pressures on the service. Some of the best research-active centres in the country are also some of the most clinically pressured environments.

Argument 4. ‘Let's leave research to the teaching hospitals and academics’. Some of the most productive centres undertaking clinical research clinicians with an enthusiasm to improve current knowledge are in district general hospitals. The extraordinary speed of the RECOVERY and other trials in the first years of COVID-19 rely very heavily on hospitals which are not principally academic centres, but have able and motivated clinicians.

Argument 5 - mainly by those who are involved in funding decisions. ‘We have to balance the books; therefore we need to cut optional extras such as research.’ Research should be seen as a long-term investment rather like capital expenditure. By investing now we significantly improve healthcare in the future. If past generations had taken an approach which sacrificed the relatively modest funding for research to plug what are almost continuous financial needs medical advances would rapidly have come to a halt. The research component of the healthcare budget in the UK is very small relative to the total but has an outsized effect when viewed over the long term.

It is of course essential that those undertaking research make it relevant to the needs of their audience. This is true of clinical papers, but also those which aim to inform policy.^5^ A paper which does not answer a question the reader is interested in will be ignored.

## Uptake of science by clinicians

New advances in science and medicine have a positive impact on patient outcomes, but only to the extent that clinicians keep abreast of current literature. This is of course difficult to do alongside all the other competing priorities. Most clinicians however take this very seriously in the UK, helped by general medical journals, specialist conferences, guideline writing groups and NICE among others. Defending the commitment to evidence-based medicine is however a necessary ongoing activity for all of us. There will always be competition for time, and many pressures, both practical and commercial to prescribe in a particular way. It is essential that as a profession we personally commit to stay up to date, but also that we support our colleagues who are involved in things such as guideline writing or medical education. It is relatively rare that practice changes based on a single paper. More commonly information is accrued over multiple studies in different centres. Guideline writing and undertaking systematic reviews are two very important things clinicians can do to support their peers.

Embracing new advances, new tools and new techniques is generally attractive to most clinicians. Less easy, and less celebrated, is letting go of older tried and tested approaches with which we have become comfortable, but now are becoming outmoded. This is however not to imply that all new advances should be taken on at the first possible opportunity. The history of medicine is littered with examples of clinicians taking up exciting but non-evidence based new options, only for them to be proven to be less effective than hoped or, in some tragic cases such as thalidomide, actively dangerous. Taking a strong scientific and evidence-based approach therefore helps us to move to new things but also protect our patients against moving too quickly before evidence of benefit and harm is properly determined.

## Scientific advice to non-clinician decision-makers

My experience of non-clinician decision-makers, including politicians from a wide variety of different political standpoints, is that *if* they choose to ask for technical advice the overwhelming majority are respectful of that scientific and clinical advice and wish to understand it. In a UK context relatively few have advanced scientific training so the advice has to be presented much as it would to an intelligent and interested patient; technical subjects can be addressed in some depth including presenting data but without prior knowledge being assumed. The only situation where political leaders tend to be (understandably) cautious is where they think a political opinion is being presented as a scientific fact.

Where senior decision-makers, including political leaders, do not understand we as the scientific and clinical technical experts have to assume it is our responsibility to adjust our way of laying out an issue to make it clearer to a nonspecialist. This is extremely common in normal medical practice and for that reason clinical scientists often have more experience of doing this than scientists from many other disciplines. Very often nonclinical leaders are having to take difficult decisions where the clinical or scientific advice is only a small part of the picture and may well not be the deciding factor. It is however essential that as professionals we present it accurately, correcting misapprehensions where we can. Most clinicians are very good at this.

What is less easy for scientists and clinicians to influence is *whethe*r technical advice is asked for in the first place. In emergencies it almost invariably is with considerable, and often misplaced, hope on the part of decision-makers that scientists or other technical experts can provide a clear ‘answer’ as to what to do.^6^ This is particular true early in an emergency, at the point that uncertainty and concern is greatest among the general public; this is also the period when technical advice and scientific data are also least certain, and decision-makers need to be made aware of this.

It is much less predictable when clinical or scientific advice will be sought between emergencies. Ensuring we are close to events, and ready to provide accurate and comprehensible responses quickly when asked maximises the chances that clinical and scientific advice will be sought appropriately and regularly. This benefits everybody.

For advice to non-clinicians it is however essential to maintain the scientific integrity of advice, and constantly to test whether it has been accurately understood. Providing an answer that is popular with the listener, but technically wrong (or overly didactic), may well help drum up repeat custom and get a clinician-scientist back into the political room, but it undermines the whole point of having scientific or clinical advice in the first place. You do not go to a doctor, lawyer or accountant to get good news but to get accurate technical advice to help you make important informed life decisions. Political decision-makers are no different in this, and the societal risks from them making decisions based on technically wrong advice are even higher. The responsibility for us as clinicians and scientists to maintain the technical integrity of the science, including highlighting uncertainties, is therefore at least as great as it is in clinical practice.

The advance of medicine has always relied on strong scientific foundations and previous scientific advances. It is definitely not necessary to be an active researcher to be a good doctor, although it is important to keep up with relevant advances where these are evidence-based. It is necessary however for us collectively as a profession to defend the integrity of our scientific discipline. Supporting those undertaking or synthesising science is essential for the future of health, and the busyness of the system should not be used as an excuse not to do this. Our predecessors were equally busy; their science is what we now rely on for current best practice. In emergencies science is often the only way out, and supporting it is if anything more important. We must maintain the integrity of our scientific advice, including its uncertainties, in giving advice to political leaders just as much as we should to individual patients.

## References

[1] Piot P, Karim SS, Hecht R, Legido-Quigley H, Buse K, Stover J, Resch S, Ryckman T, Møgedal S, Dybul M, Goosby E. (2015). Defeating AIDS—advancing global health. Lancet.

[2] Pradelle A, Mainbourg S, Provencher S, Massy E, Grenet G, Jean-Christophe LE (2024). Deaths induced by compassionate use of hydroxychloroquine during the first COVID-19 wave: an estimate. Biomed Pharmacother.

[3] You D, Hug L, Ejdemyr S, Idele P, Hogan D, Mathers C, Gerland P, New JR, Alkema L. (2015). Global, regional, and national levels and trends in under-5 mortality between 1990 and 2015, with scenario-based projections to 2030: a systematic analysis by the UN Inter-agency Group for Child Mortality Estimation. Lancet.

[4] British Hearth Foundation Factsheet 2024. https://www.bhf.org.uk/-/media/files/for-professionals/research/heart-statistics/bhf-cvd-statistics-uk-factsheet.pdf Accessed 12/02/2024.

[5] Whitty CJM. (2015). What makes an academic paper useful for health policy?. BMC Med.

[6] Delfraissy JF, Horgan M, Mølbak K, Simón FS, Stadler T, van Dissel J, Van Gucht S, Ricciardi W, Wieler L, Vallance P, Atlani-Duault L. (2024). Scientific advisory councils in the COVID-19 response. Lancet.

